# COVID-19 lockdown impact on the physical activity of adults with progressive muscle diseases

**DOI:** 10.1136/bmjno-2021-000140

**Published:** 2021-03-18

**Authors:** Sarah F Roberts-Lewis, Mark Ashworth, Claire M White, Michael R Rose

**Affiliations:** 1 School of Population Health and Environmental Sciences, King's College London, London, UK; 2 Neurology, King s College Hospital, London, UK

**Keywords:** COVID-19, muscle disease, muscular dystrophy, rehabilitation, physiotherapy

## Abstract

**Introduction:**

This short article summarises findings about reduced physical activity of adults with progressive muscle disease as a result of COVID-19 lockdown.

**Methods:**

As part of an ongoing longitudinal cohort study, we prospectively and objectively measured physical activity using accelerometry at baseline in 2019 and follow-up in 2020. A subset of 85 participants incidentally had follow-up data collected during the first UK COVID-19 lockdown from 23 March to 4 July 2020. Thus, for this cohort we had activity data from before and during the COVID-19 pandemic and we were able to prospectively and accurately quantify the changes in their physical activity.

**Results:**

Our data highlighted reduced overall activity intensity and reduced light activity time in particular.

**Conclusions:**

From our findings, we can infer specific evidence-based recommendations about how to redress inactivity secondary to COVID-19 restrictions for adults with progressive muscle diseases. These recommendations are likely to be generalisable to other groups who are vulnerable to functional decline secondary to prolonged inactivity.

## Introduction

The COVID-19 pandemic led to ‘lockdown’ periods and ongoing restrictions to usual activities in many countries. People with neuromuscular diseases are at particular risk of functional decline secondary to prolonged inactivity.^1^ For vulnerable groups, there is little evidence-based public health advice about how to redress physical activity deficits resulting from COVID-19 restrictions. Serendipitously, our ongoing, longitudinal cohort study of adults with progressive muscle diseases, prospectively and objectively measured physical activity before and during the COVID-19 crisis (in 2019 and 2020 respectively). In analysing this subset of our data, we aimed to quantify activity changes resulting from COVID-19 lockdown. Thereby, we aimed to make informed recommendations to mitigate activity deficits during and after COVID-19 restrictions, for adults with progressive muscle diseases. People with other long-term conditions might also benefit from such advice.

## Methods

For this prospective cohort study, remote, home-based physical activity baseline data were collected from April to September 2019 with follow-up 9 months later in 2020. A subsample of participants had follow-up data collected during the UK COVID-19 lockdown from 23 March until fourth July 2020. Participants were adults with confirmed diagnoses of muscular dystrophy and other inheritable myopathies recruited via Muscular Dystrophy UK advertisement and patient registries at The John Walton Muscular Dystrophy Research Centre, Newcastle University. The primary outcome was overall activity intensity measured in mean accelerations per minute using a GENEActiv tri-axial accelerometer (Activinsights, Kimbolton, UK). Secondary outcomes included accelerometer-derived activity frequency and activity time at inactive, light, moderate and vigorous intensity.

Participants consented to the study and wore the accelerometer continuously for a week at baseline and follow-up. It sampled at 10 Hz and data were processed in 1 min epochs in milligravitational units (milli-g) with gravitational correction using the GGIR package in R V.3.6.0.^2^ Activity frequency was the percentage per day of hourly, non-consecutive activity of 80 milli-g/min for ≥5 min each hour between 09:00 to 17:00 daily. Intensity cut-points were light ≥30 milli-g/min, moderate ≥100 milli-g/min and vigorous activity ≥400/milli-g/min. These yielded time in minutes of inactivity, light activity and bouts of ≥10 min of moderate and vigorous activity.

Questionnaire data collected at baseline and follow-up included the Health Assessment Questionnaire (HAQ).^3^ It measures disability from 0 to 3 (‘without any difficulty’ to ‘unable to do’) for 24 items including dressing, grooming, arising, eating, walking, hygiene, reach, grip, errands, chores and any aids, devices or help from a person required for each activity.

Changes in accelerometer outcomes and HAQ from baseline to follow-up were examined using mean difference and paired t-test (or non-parametric equivalents); alpha was set at 0.05. A retrospective power calculation was performed for the lockdown subsample, and a low (0.20) to moderate (0.50) effect size of activity reduction was expected.

## Results

There was a mean of 9.8 months between baseline and follow-up. Full data were collected for 103 participants at baseline; three were lost to follow-up. Of the 100 participants with both baseline and follow-up data, ages ranged from 18 to 81 years (mean=48 years). Diagnoses included limb girdle muscular dystrophy (33), facioscapulohumeral dystrophy (24), inclusion body myositis (16), dystrophinopathy (Becker/Duchene including manifesting female carriers) (10), myotonic dystrophy (9) and congenital myopathies (including centronuclear, Bethlem, Ullrich, SEPN1-related and Emery Dryfuss) (8). There were 41 wheelchair users. Fifteen participants had follow-up data collected before lockdown; 85 were followed up during lockdown. All participants had at least 5 days of useable accelerometry data at baseline and follow-up. There was no missing HAQ data.

**Figure 1:**
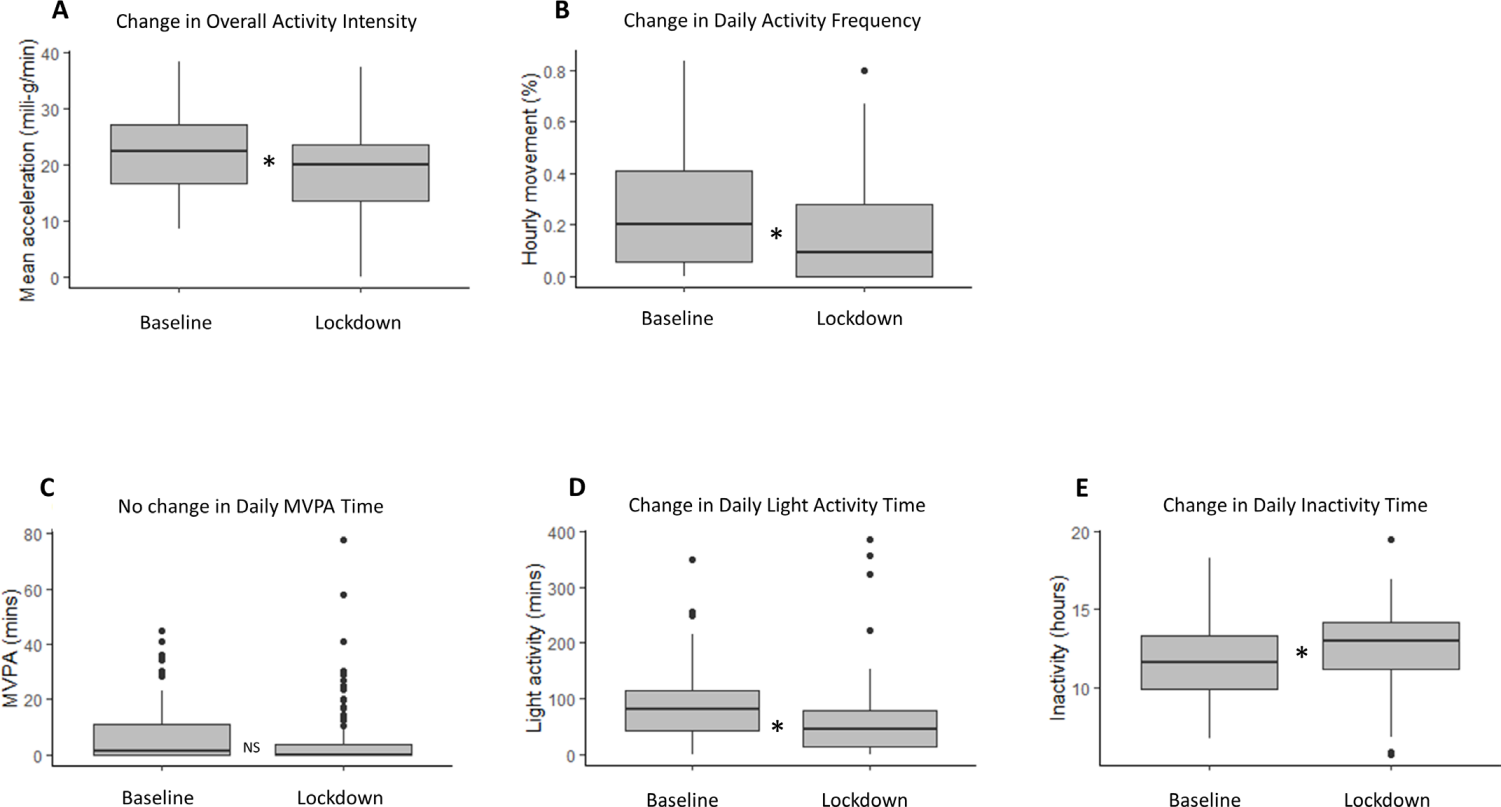
Box plots illustrating activity changes from baseline to lockdown follow-up (N=85). (A) *t=3.93, df=84, p=0.0002, mean difference=3.3 milli-g/min, 95% CI 1.6 to 5.0. (B) *Wilcoxon signed rank p<0.0001, median difference=11.1%, IQR 3.7–14.8. (C) NS Wilcoxon signed rank p=0.0670, median difference=1.2 min/day, IQR 0.0–2.8. (D) *t=3.10, df=84, p=0.0027, mean difference=25.2 min/day, 95% CI 9.0 to 41.4. (E) *t=−4.08, df=84, p=0.0001, mean difference=−0.9 hours/day, 95% CI −0.5 to −1.4. MVPA moderate and vigorous physical activity; NS not significant.

Participants had generally low physical activity levels at baseline (for the lockdown subsample, overall intensity of 21.9 mean accelerations per minute (SD 7.0)). During lockdown, there was a significant reduction in overall activity intensity (see figure 1). The effect size was 0.47 and the retrospective power calculation was 95%.

At baseline, all participants were generally inactive. The lockdown subsample (n=85) was inactive for a mean of 11.8 hours/day (SD 2.4) at baseline. They did a mean of 84.5 min/day (SD 66.0) of light activity and had a low frequency of hourly movement (median=20.4%, IQR 5.6–40.7). Most did little moderate and vigorous physical activity (MVPA) (median 1.2 min/day, IQR 0.0–11.0). During lockdown, inactivity increased significantly by a mean of 55 min/day. Light activity time significantly reduced by a mean of 25 min/day and frequency of hourly movement reduced by a median of 11%. However, there was no significant change in MVPA time. No significant effect of diagnostic group on activity changes was detected. For the non-lockdown subsample (n=15), there was no significant change in any physical activity outcomes from baseline to follow-up.

At baseline, the median HAQ disability index was 1.81 (IQR 0.97–2.50). There was no significant difference in HAQ disability index between lockdown and non-lockdown subsamples. For the lockdown subsample, there was no significant worsening in disability from baseline to follow-up, although for the non-lockdown subsample, there was a small, but significant reduction in the HAQ disability index (t=3.59, df=14, p=0.003, mean difference=0.17, 95% CI 0.07 to 0.28).

## Discussion

COVID-19 lockdown adversely affected the physical activity of adults with progressive muscle disease. A key finding of our study was that light activity time and frequency of movement during the day were significantly reduced. Somewhat surprisingly, MVPA time was largely unaffected during lockdown; this might be explained by low baseline levels of MVPA.

Our findings are similar to other studies reporting reduced physical activity during lockdown; for people with neuromuscular diseases, self-reported vigorous activity was unchanged, while moderate activities and walking reduced significantly.^4^ However, the mean change in daily MVPA was considerably lower in our study (1.2 min compared with self-report of ~21 min/day), and we found significant reductions in light activity only. Self-report activity measures are not sufficiently accurate or comprehensive to capture light activity or frequency of daily activity detected by accelerometry. Moreover, the discrepancy in MVPA time could be explained by self-report overestimation, for example, reporting light activities as being moderate.

The reduced light activity time and frequency of daily movement detected in our study resulted from restrictions on leisure activities and the loss of brief, cumulative, active minutes which usually intersperse pursuits such as socialising and work. As these pursuits are not exercise-focused, it might be difficult for individuals to detect such subtle light activity loss. However, light activity and regular movement throughout the day are associated with improved health outcomes.^5^ And the 2020 WHO activity guidelines stress that ‘every move counts towards better health’.^6^ Thus, it is important to redress light activity reduction during and after lockdowns, especially with ongoing COVID-19 restrictions. Reduced incidental and cumulative light activity could be replaced with extra light activity, such as t′ai chi, yoga, pilates, chair exercises, gentle stretching, bathing and slow walking (examples from WHO activity recommendations for people living with disability).^6^ Appropriate light activity would lessen the impact of inactivity disuse on functional muscle groups, including those required for ambulation and core stability.

The key strength of this study is the prospective design and objective accelerometer data, which are more comprehensive and accurate than self-reported physical activity measurement.^7^ The study could have been improved by including greater numbers per muscle disease diagnostic category and a larger non-lockdown comparator group because there is a risk of type II error in the null results. However, in slowly progressive conditions, disease worsening would not usually account for clinically significant functional deterioration in less than a year.^8 9^ Furthermore, the lack of activity change in the non-lockdown subsample, combined with their slightly worsened disability, compared with the reduced activity in the lockdown subsample, without significantly changed disability, are all suggestive that lockdown was associated with reduced physical activity.

Based on our unique objective activity data, we recommend a minimum of 30 min of extra light activity per day and 5 min of movement each hour throughout the day to redress the impact of COVID-19 inactivity for adults with progressive muscle diseases. This evidence-based activity advice could be generalised to other groups, especially those vulnerable to deterioration resulting from prolonged inactivity, including people with other long-term conditions, neuromuscular diseases and disabilities, and the elderly. Our advice represents an easily disseminated public health message.
